# Predicting fluid responsiveness in spontaneously breathing parturients undergoing caesarean section via carotid artery blood flow and velocity time integral measured by carotid ultrasound: a prospective cohort study

**DOI:** 10.1186/s12884-024-06246-z

**Published:** 2024-01-12

**Authors:** Shaobing Dai, Chun Wang, Xia Tao, Jianjun Shen, Lili Xu

**Affiliations:** 1 Department of Anaesthesiology, Women’s Hospital, Zhejiang University School of Medicine, Hangzhou, Zhejiang Province China; 2 Department of Ultrasound, Women’s Hospital, Zhejiang University School of Medicine, Hangzhou, Zhejiang Province China; 3 Department of Anaesthesiology, the Second Affiliated Hospital, School of Medicine, Zhejiang University, Hangzhou, Zhejiang Province China; 4 Zhejiang Provincial Clinical Research Center for Obstetrics and Gynecology, Hangzhou, Zhejiang Province China

**Keywords:** Carotid artery blood flow, Velocity time integral, Ultrasonography, Fluid responsiveness

## Abstract

**Background:**

Present evidence suggests that the Doppler ultrasonographic indices, such as carotid artery blood flow (CABF) and velocity time integral (VTI), had the ability to predict fluid responsiveness in non-obstetric patients. The purpose of this study was to assess their capacity to predict fluid responsiveness in spontaneous breathing parturients undergoing caesarean section and to determine the effect of detecting and management of hypovolemia (fluid responsiveness) on the incidence of hypotension after anaesthesia.

**Methods:**

A total of 72 full term singleton parturients undergoing elective caesarean section were enrolled in this study. CABF, VTI, and hemodynamic parameters were recorded before and after fluid challenge and assessed by carotid artery ultrasonography. Fluid responsiveness was defined as an increase in stroke volume index (SVI) of 15% or more after the fluid challenge.

**Results:**

Thirty-one (43%) patients were fluid responders. The area under the ROC curve to predict fluid responsiveness for CABF and VTI were 0.803 (95% CI, 0.701–0.905) and 0.821 (95% CI, 0.720–0.922). The optimal cut-off values of CABF and VTI for fluid responsiveness was 175.9 ml/min (sensitivity of 74.0%; specificity of 78.0%) and 8.7 cm/s (sensitivity of 67.0%; specificity of 90.0%). The grey zone for CABF and VTI were 114.2-175.9 ml/min and 6.8–8.7 cm/s. The incidence of hypotension after the combined spinal-epidural anaesthesia (CSEA) was significantly higher in the Responders group 25.8% (8/31) than in the Non-Responders group 17.1(7/41) (*P* < 0.001). The total incidence of hypotension after CSEA of the two groups was 20.8% (15/72).

**Conclusions:**

Ultrasound evaluation of CABF and VTI seem to be the feasible parameters to predict fluid responsiveness in parturients undergoing elective caesarean section and detecting and management of hypovolemia (fluid responsiveness) could significantly decrease incidence of hypotension after anaesthesia.

**Trial registration:**

The trial was registered at the Chinese Clinical Trial Registry (ChiCTR) (www.chictr.org), registration number was ChiCTR1900022327 (The website link: https://www.chictr.org.cn/showproj.html?proj=37271 ) and the date of trial registration was in April 5, 2019. This study was performed in accordance with the Declaration of Helsinki and approved by the Research Ethics Committee of Women’s Hospital, Zhejiang University School of Medicine (20,180,120).

## Introduction

Over the past decades, static and dynamic assessment of volume status and fluid reactivity has extremely contributed to perioperative fluid management and enhanced recovery after surgery and has become an important tool in the diagnosis and treatment of obstetric anaesthesia [1]. Among them, the widespread use of stroke volume change (SVV) was limited by its invasiveness and application conditions, such as complete muscle relaxation, mechanical ventilation, and absence of arrhythmia [2]. Currently, because Doppler ultrasound can show the blood flow and internal structure of the heart and the large blood vessels in real time, many ultrasound variables are introduced to evaluate the volume status and guide fluid resuscitation [3, 4]. Unfortunately, the widespread use of inferior vena cava collapsibility index (IVCCI) to predict fluid reactivity during caesarean section was prevented by the pregnant uterus compression, adjacent to surgical site, and the technical difficulty in ultrasound identifying. Interestingly, some previous studies have confirmed that parameters measured by carotid ultrasound, such as corrected flow time, can well predict volume responsiveness in spontaneously breathing patients [5, 6]. Importantly, emerge evidence further demonstrated that changes in cardiac preload and cardiac output translate directly into changes in carotid blood flow, supporting the possibility that carotid blood flow can be a proxy for cardiac output [7]. Additionally, carotid artery blood flow has been suggested as a better indicator of cardiac output and was less affected by measurement problems than corrected carotid flow time [8].

As is known to all, there are various significant changes in hemodynamic during pregnancy, including blood volume, heart rate, stroke volume (SV), cardiac output (CO), vascular resistance, and colloid osmotic pressure [9]. These changes affect maternal and fatal oxygen transport, including oxygen affinity, delivery, and consumption [10]. In addition, axial anaesthesia during caesarean section often causes sympathetic nerve block, increased venous volume, and decreased systemic vascular resistance (SVR), eventually leading to relatively low blood volume and even decompensated hypotension. Therefore, predicting intraoperative fluid responsiveness is an important focus, as improper fluid treatment can lead to adverse outcomes [10]. However, it is still unclear whether VTI and CABF measured by ultrasound can be used as the variables of intravascular volume status and whether they have a certain guiding role in perioperative fluid therapy for spontaneous breathing parturients. The purpose of this present study was to evaluate the predictive power of ultrasound measurement of CABF and VTI for fluid responsiveness in parturients undergoing elective caesarean section and to determine the effect of detecting and management of hypovolemia (fluid responsiveness) on the incidence of hypotension after anaesthesia.

## Methods

### Design, setting, and participants

A single-centre prospective cohort study was conducted at grade A tertiary hospital which was a large-scale obstetrics and gynaecology hospital in China. We collected the perioperative data of all patients who underwent elective caesarean section between April 2019 and May 2019.

In our study, we recruited seventy-eight American Society of Anaesthesiologists (ASA) Class I-II parturients with elective caesarean section. Women over 18 years of age who undergo routine prenatal examinations for a full-term single pregnancy and women over 37 weeks of gestation were included. Women with hypertension, preeclampsia, undergoing emergency caesarean section and women with a history of chronic cardiopulmonary diseases as well as liver or kidney failure were excluded.

### Study procedures

Preoperative women with elective surgery routinely fast for 8 h and no drinking for 2 h. No premedication was administered. Upon arrival in the operating theatre, peripheral venous access was established using an 18 G intravenous cannula inserted into an upper limb vein. After the parturient entered the operating room, standard monitoring, including non-invasive blood pressure (NIBP), heart rate, pulse oximetry, and electrocardiography, was applied.

The parturients underwent carotid ultrasound and transthoracic echocardiography separately. The ultrasound-guided predictive measurements of fluid responsiveness included CABF, VTI, stroke volume index (SVI), and hemodynamic parameters. They were measured before and five minutes after receiving a fluid challenge of 6% hydroxyethyl starch (130/0.4) 6 ml/kg ideal body weight [(height cm-70) *60%] over 10 min. Fluid responsiveness was determined by a 15% or more increase in SVI after fluid challenge by transthoracic ultrasound. According to fluid responsiveness, the patients were divided into two groups: the Non-Responders group and the Responders group. The patients were stable during the measurement period and did not receive any vasoactive drug therapy.

All patients received the combined spinal-epidural technique. Combined spinal-epidural anaesthesia(CSEA) was administered at the L3-4 interspace under local anaesthesia, with patients positioned in the left lateral position. After confirming the presence of clear cerebrospinal fluid (CSF), 15 mg of hyperbaric ropivacaine were immediately injected intrathecally over a duration of 30 s. An epidural catheter was then inserted 4-5 cm into the epidural space. Subsequently, the patient was placed in a supine position with a wedge placed under the right buttock, and oxygen was delivered at a rate of 2 L/min through a nasal catheter.

During the aesthetic operation, fluid management differed between the two groups [11]. A co-load of warmed lactated Ringer’s solution at a volume of 10 ml/kg was infused over a period of 15–20 min in the Responders group and patients received warmed lactated Ringer’s solution continuous infusion of 8 mg/kg/h in the Non-Responders group during CSEA [12, 13]. After completion of the intrathecal injection, hypotension after CSEA was recorded, and the incidence of hypotension was calculated. Hypotension was defined as systolic blood pressure < 90 mmHg or < 80% of baseline and was treated with i.v. 4ug noradrenaline [11].

### Carotid ultrasonography

**Fig. 1:**
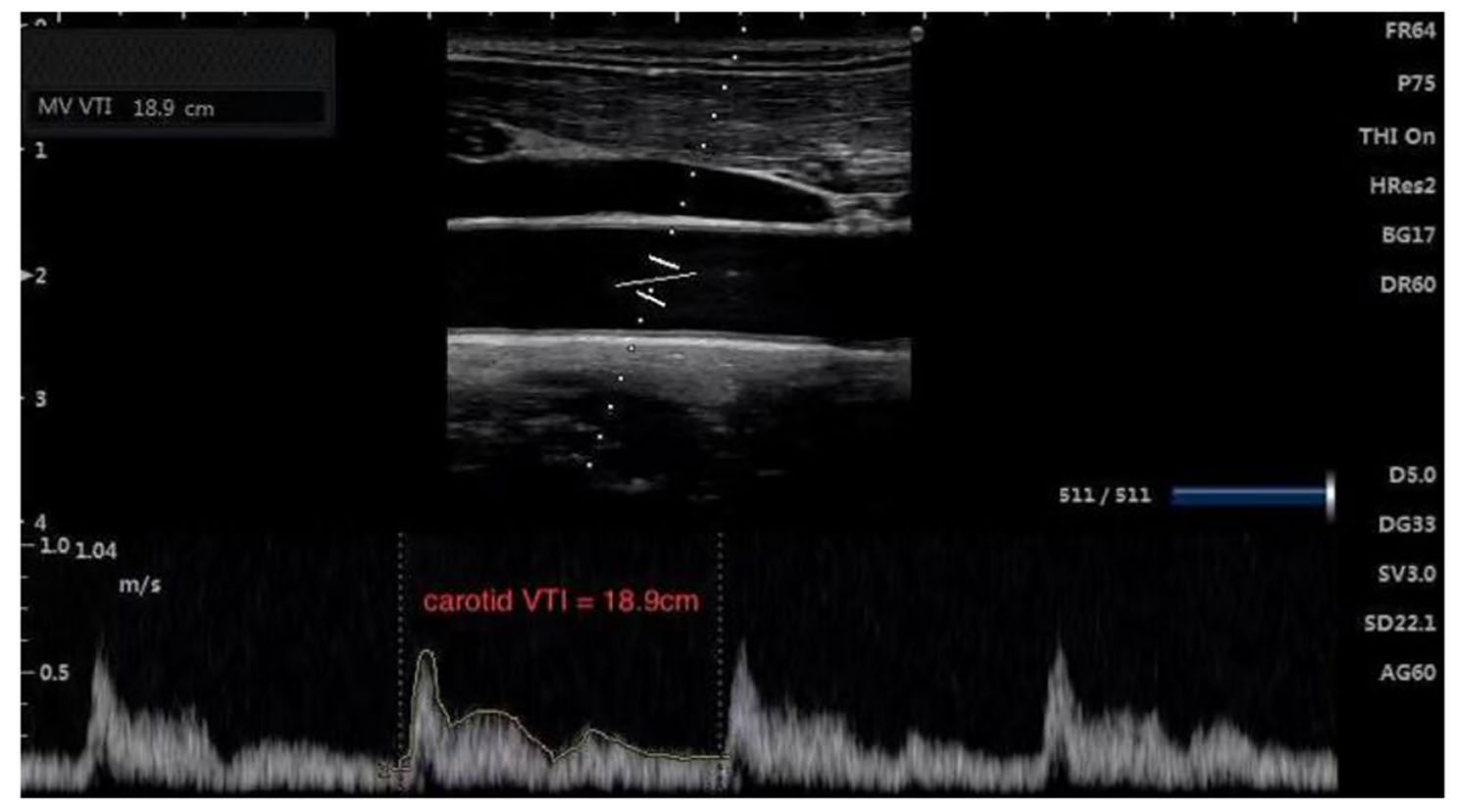
CAD and VTI

CABF and VTI were measured by two anaesthesiologists (Chun Wang and Jianjun Shen) with specialized ultrasound training. Prior to the study, they had completed carotid ultrasonography in 50 patients and were approved by sonographers. We placed the patients in the supine position with the images of carotid artery diameter and VTI obtained by SONIMAGE HS1 (KONICA MINOLTA Inc, Shanghai, China). The 6–13 MHz variable frequency probe was placed vertically in the neck, with the marker pointing to the patients’ head. The long axis B-mode image of the right common carotid artery was located at the lower margin of the thyroid cartilage. The sampling line was placed at the centre of the carotid lumen, about 2 cm from the bifurcation. The area under the pulse Doppler tracer VTI curve of carotid blood flow obtained by angle correction was determined by the automatic tracking of monopulse waveform. (Fig. 1). Carotid artery diameter and VTI were measured three times by the examiner and averaged for analysis. The ultrasound software automatically calculated the carotid blood flow per minute, and the formula is: carotid artery blood flow (mL/min) =πx(carotid artery diameter/2)2 x (VTI) x (60 s) [8].

### Cardiac ultrasonography

The measurement of cardiac stroke volume is performed by a professional ultrasound doctor (Xia Tao). The parturient was placed in the left lateral decubitus position and 1.5–4.5 MHZ phased array probe was the used for SV examination. The diameter of the left ventricular outflow tract at the systolic aortic apex was measured by parasternal long axis echocardiography. The area of left ventricular outflow tract was calculated as π x (the square of the left ventricular outflow tract radius) [14]. Aortic blood flow VTI was calculated from the area under the pulse-wave Doppler signal envelope obtained from the apical five-chamber cutting surface at the level of the aortic ring and was determined by the average value of five consecutive pulses in a complete respiratory cycle. SVI was calculated as (left ventricular outflow tract area x aortic flow VTI)/body surface area (BSA), and BSA was calculated as BSA(m^2^) = 0.0061×body length (cm) + 0.0128×body weight (kg)-0.1529 [15].

### Study endpoints

The primary endpoint was to determine the predictive value of carotid artery blood flow and VTI for fluid responsiveness (≥ 15% increases in SVI after fluid challenge) in spontaneous breathing parturients and the effect of detecting and management of hypovolemia (fluid responsiveness) on the incidence of hypotension after anaesthesia [16].

### Statistical analysis

SPSS 23.0 (Chicago, IL, USA) was used for data statistical analysis. The study used PASS software 15.0 for sample size calculation. The results of a previous study showed an area under the ROC curve for carotid VTI to predict fluid responsiveness was 0.869 [17]. The area under the receiver operating characteristic (ROC) curve for VTI to predict fluid responsiveness was 0.83 and the incidence of the Responders group was 45% in our pre-experiment. We assumed an area under the ROC curve of 0.8 and an incidence of 40% in the responder group, which is lower than that in the pre-experiment and the previous study, with a power of 0.9 and a two-sided type I error of 0.05, and the research needed to include 63 subjects, taking into account a 10% dropout rate. A total of 72 women undergoing elective caesarean section were finally included in the study. Normality of the data distribution was assessed using Kolmogorove-Smirnov and Shapiroe-Wilk tests. Continuous variables were expressed as mean (standard deviation) if data were normally distributed or median (interquartile range) if not. Categorical variables were expressed as absolute number (%). Responder and non-responder groups were compared with a paired t-test for normally distributed data, ManneWhiney U-test for non-normally distributed data, and X^2^ test or Fisher’s exact test, as appropriate, for categorical variables.

The ROC curve was applied to discern the predictive ability of indicators. Area under the curve (AUC) provides a global measure of measurement accuracy. The guidelines recommend that 0.5 < AUC ≤ 0.7 represents low accuracy, 0.7 < AUC ≤ 0.9 suggests moderate accuracy, and 0.9 < AUC ≤ 1.0 stands for high accuracy. An AUC higher than 0.75 is considered good. We calculated the 95% confidence interval (CI), and *p* < 0.05 was considered as statistical significance. The “optimal” cut-off values were assessed by using maximizing Youden’s index (J = Sensitivity + Specificity-1 = Sensitivity-False-Positive Rate) [18]. The cut-off values defining the gray area was determined by a correlation value of 90% sensitivity and 90% specificity [19]. Importantly, the intra-observer variability (repeatability) and inter-observer variability (reproducibility) were evaluated in all patients of assessments of carotid artery blood flow and VTI. Variability was tested by dividing the absolute difference between the two values by their average value. Accordingly, the inter-observer reproducibility for carotid artery blood flow and VTI was also recognized in all data sets by calculating a coefficient of variation (CV) and an intraclass correlation coefficient (ICC). Bland-Altman plot was applied to test the inter-observer agreement in estimating carotid artery blood flow and VTI. A *P*-value < 0.05 (two-tailed) was considered statistically significant.

## Results

### Participants and flow diagram

**Fig. 2:**
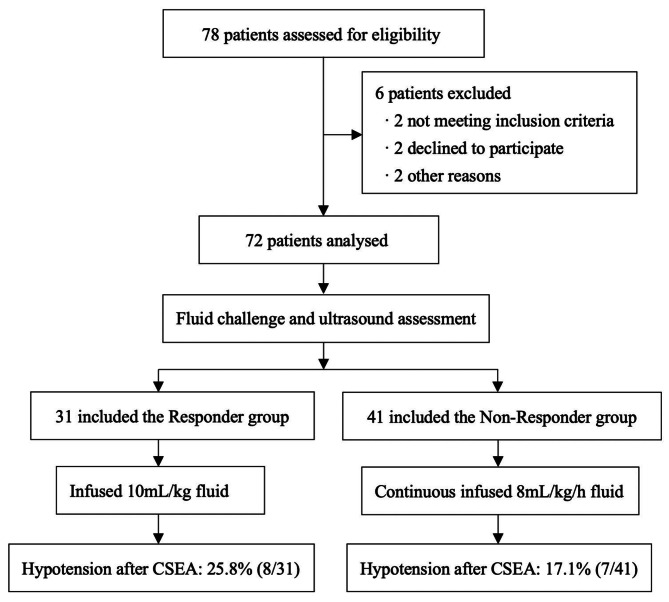
The process of subject selection

Of the 78 patients assessed for eligibility, 6 were excluded because of not meeting inclusion criteria (*n* = 2), declined to participate (*n* = 2), and other reasons(*n* = 2). Therefore, 72 subjects were enrolled in the final analysis (Fig. 2). The main characteristics of the subjects were comparable between responders (*n* = 31) and non-responders (*n* = 41) (Table 1). There were no significant differences in patient characteristics between groups (*p* > 0.05) (Table 1).

**Table 1:** Patient characteristics

	Responders group (*n* = 31)	Non-responders group (*n* = 41)	*P* value
Age (yr)	32.9 ± 3.5	34.0 ± 4.8	0.265
ASA (I/II)	20/11	31/10	0.305
Height (cm)	159.7 ± 5.6	159.8 ± 5.7	0.923
Weight (kg)	68.7 ± 8.0	67.9 ± 8.2	0.701
BMI	26.9 ± 3.1	26.6 ± 2.5	0.557
Duration of Surgery (min)	49.2 ± 14.5	52.2 ± 20.1	0.482

Values are numbers or means ± SD.

*p < 0.05 compared with Responders group

BMI: Body mass index (kg/m^2^); ASA: American Society of Anaesthesiologists

### Haemodynamic variables before and after fluid challenge

**Fig. 3:**
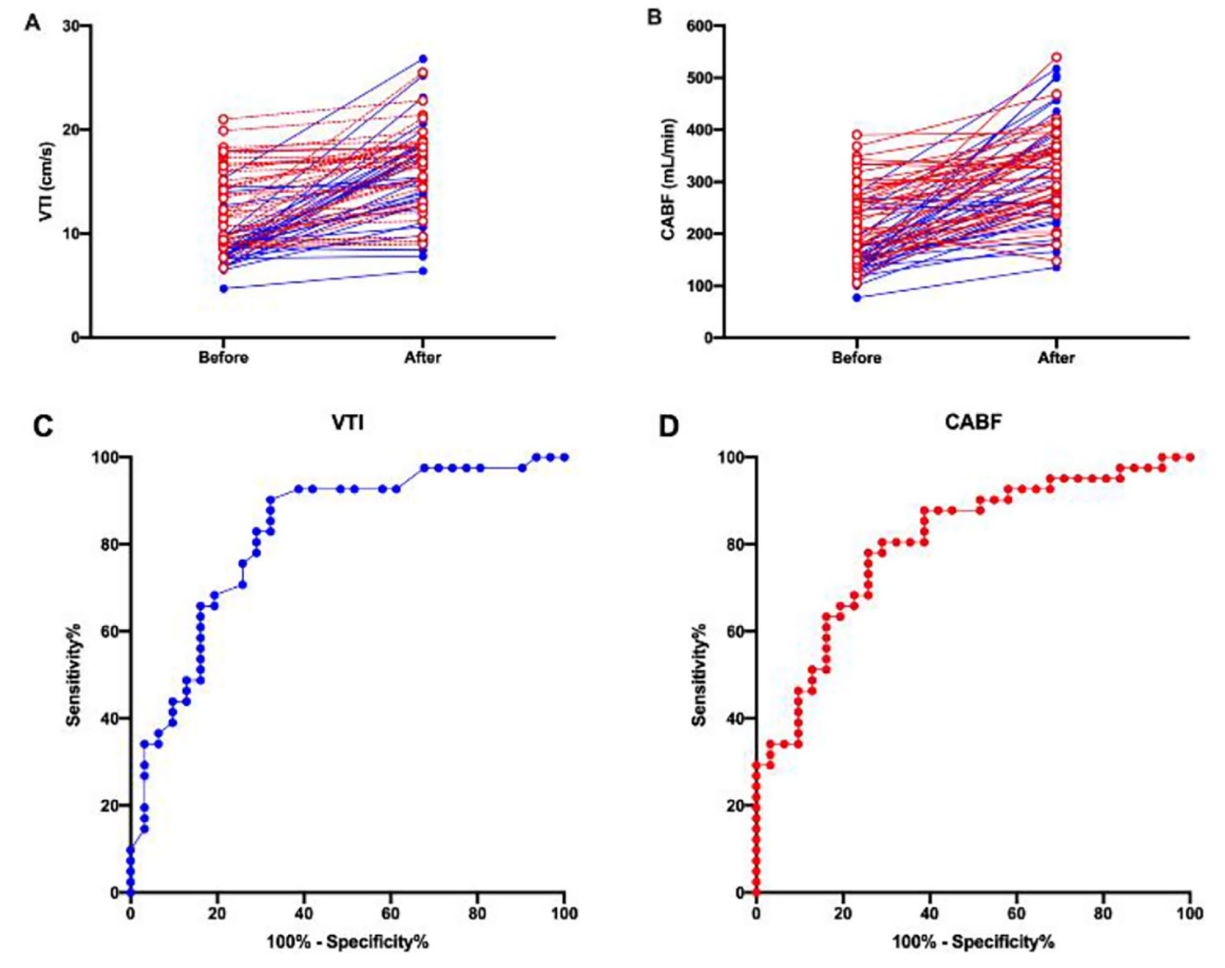
Individual responses to fluid challenge and ROC curve for VTI and CABF

Fluid challenge markedly increased SVI, carotid artery blood flow, and VTI in both the Responders and the Non-Responders group (*p* < 0.05). (Table 2) (Fig. 3). Before fluid challenge, SVI, VTI, and carotid artery blood flow was obviously lower in responders than in non-responders (*p* < 0.05) (Table 2). In contrast, after fluid challenge, SVI, VTI, and carotid artery blood flow were all not significantly different between the two groups (Table 2). Both MAP and HR were not significantly different between the two groups before and after the fluid challenge (Table 2). The incidence of hypotension after the CSEA was significantly higher in the Responders group 25.8% (8/31) than in the Non-Responders group 17.1(7/41) (*P* < 0.001). The total incidence of hypotension after CSEA of the two groups was 20.8% (15/72).

**Table 2:** Hemodynamic variables before and after fluid challenge

	Responders group (*n* = 31)		Non-responders group (*n* = 41)		*P* value	*P* value
	Before	After	Before	After	Before	After
CABF (ml/min)	161.2 ± 50.4	317.3 ± 105.1*	236.4 ± 72.9#	321.7 ± 79.4*	0.0002	0.843
**VTI (cm/s)**	**9.0 ± 2.9**	15.8 ± 4.8*	**13.1 ± 3.9#**	16.4 ± 3.7*	**0.0003**	**0.587**
**SVI (ml m** ^**− 2**^ **)**	**61.7 ± 11.2**	84.5 ± 16.0*	**68.3 ± 13.2#**	79.5 ± 16.4*	**0.018**	**0.196**
**HR (beat min-1)**	**87.5 ± 14.3**	**88.2 ± 13.5**	**84.4 ± 11.7**	**83.2 ± 11.6**	**0.318**	**0.096**
**MAP (mmHg)**	**83.7 ± 7.5**	**89.3 ± 8.6**	**85.3 ± 14.9**	**90.8 ± 8.4**	**0.573**	**0.473**

The data are reported as mean ± SD

*p < 0.05 compared with before fluid challenge. #*p* < 0.05 compared with Responders group

SVI: stroke volume index; VTI: carotid artery velocity time integral; CABF: carotid artery blood flow

MAP: Mean arterial pressure; HR: Heart rate

### The ability of carotid artery blood flow and VTI to predict fluid responsiveness

The area under the ROC curve to predict fluid responsiveness for carotid artery blood flow was 0.803 (95% CI, 0.701–0.905) and for VTI was 0.821 (95% CI, 0.720–0.922) (Fig. 3). The sensitivity and specificity for carotid artery blood flow and VTI were 74%, 78% and 67%, 90%. Their cut-off values are 175.9 ml/min and 8.7 cm/s. The positive and negative predictive values for CABF and VTI were 0.72, 0.67 and 0.84, 0.79 (Table 3).

**Table 3:** Prediction of fluid responsiveness by receiver operating characteristic curves of the baseline VTI and CABF

	AUROC curve (95% CI)	*P*-value	Optimal cut-off value	Grey zone	Patients in grey zone (%)		Sensitivity (%) (95% CI)	Specificity (%) (95% CI)	Youden index	PPV (%) (95%CI)	NPV (%) (95%CI)
VTI	0.821 (0.720–0.922)	0.0003	8.7 cm/s	6.8–8.7 cm/s		13(18%)	67.0(50.1–86.0)	90.0 (80.0-100.0)	0.577	0.84 (0.68-1.00)	0.79 (0.66–0.91)
CABF	0.803 (0.701–0.905)	0.0001	175.9 ml/min	114.2-175.9 ml/min		29(40%)	74.0(57.0–91.0)	78.0 (64.0–92.0)	0.520	0.72 (0.55–0.89)	0.67 (0.67–0.93)

VTI: carotid artery velocity time integral;CABF: carotid artery blood flow; AUROC: area under the receiver operating characteristic; CI, confidence interval; PPV: positive predictive values; NPV: negative predictive values

* Optimal cut-off values were determined by maximizing the Youden index

CAD and VTI at 2 cm proximal to the carotid bulb were measured

Among the 78 patients who met the inclusion criteria, 6 were excluded, 72 were agreed by consent, 72 were studied, 31 were in the Responders group and 41 were in the Non-Responders group

SVI: stroke volume index; CSEA: combined spinal-epidural anaesthesia

Upper row: individual responses to fluid challenge for VTI(A) and CABF(B). Responders are presented as blue full line and closed circles; Non-responders are presented as red dashed line and open circles

Lower row: receiver operating characteristic curves showing the ability of VTI (C) and CABF (D) before fluid challenge to discriminate responders and non-responders

The areas under the curves for VTI and CABF were 0.802 (95% confidence interval 0.706–0.898), 0.812 (95% confidence interval 0.714–0.909), and 0.846 (95% confidence interval 0.762–0.930), respectively

Responders are represented by blue full lines and closed circles; Non-responders are represented by red dashed lines and open circles

VTI: carotid artery velocity time integral;CABF: carotid artery blood flow

Red dotted lines indicate the mean difference (bias), and black dotted lines indicate the 95% limits of agreement (1.96 x standard deviation)

### The inter-observer agreement in estimating VTI and carotid artery blood flow

**Fig. 4:**
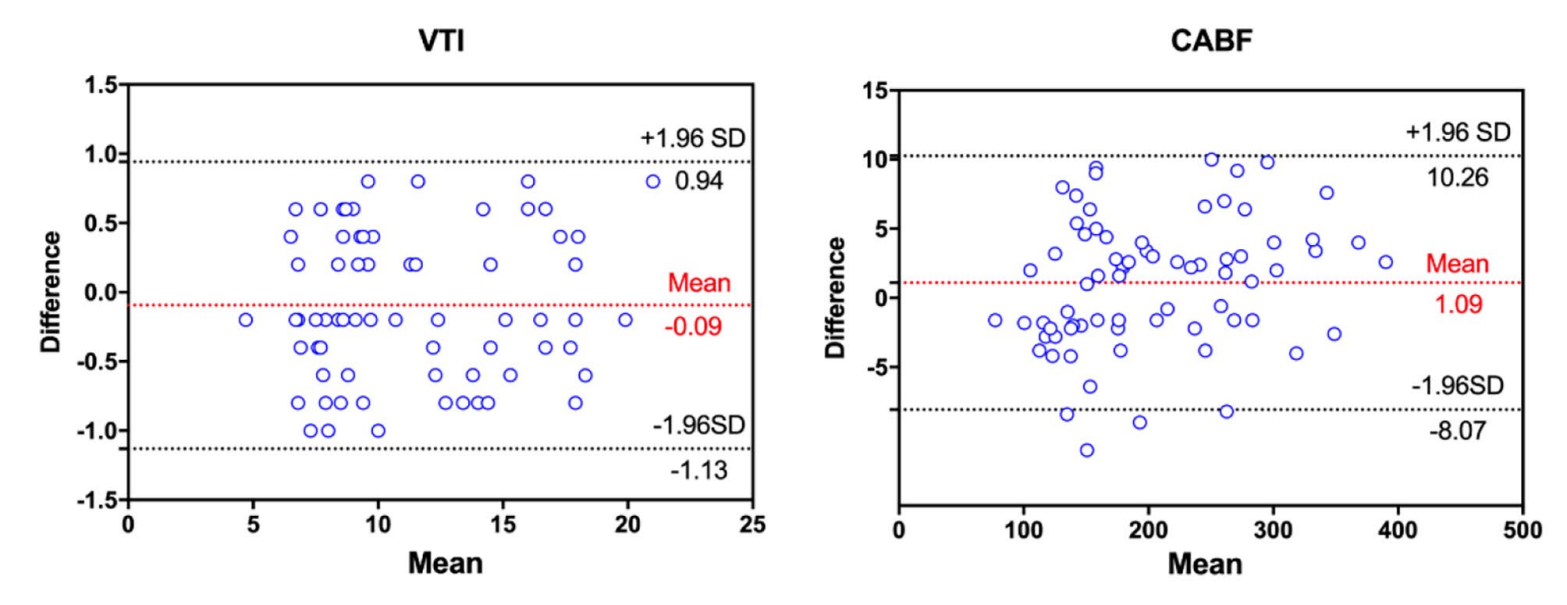
BlandeAltman plots for inter-observer agreement of VTI and CABF.

For VTI measurements, intra-observer variability and inter-observer variability were 4.1 (2.6) % and 4.6 (2.9)%, respectively. For carotid artery blood flow measurements, inter-observer variability was 2.3 (2.0) % and 2.2 (1.6)%, respectively. Inter-observer reproducibility for estimating VTI was excellent, with an ICC of 0.992 (95% CI, 0.988–0.994) and a CV of 35.5%. Inter-observer reproducibility for estimating carotid artery blood flow was also excellent, with an ICC of 0.998 (95% CI, 0.997–0.999) and a CV of 36.2%. Using Bland-Altman analysis for evaluating inter-observer agreement in estimating VTI and carotid artery blood flow, the mean biases were − 0.09 ms [with 95% limits of agreement (LOA) between − 1.13 and 0.94 ms] and 1.09 (with 95% LOA between − 8.07 and 10.26), respectively (Fig. 4).

## Discussion

The assessment of circulating capacity and rehydration needs is the basis for recovery, but remains largely empirical. Recently, several dynamic indices relying on arterial Doppler ultrasonography have been used to assess preoperative intravascular volume status in mechanically ventilated patients. However, current studies on dynamic indicators for evaluating spontaneous fluid response have often been disappointing, often due to a number of objective and subjective factors, including cardiopulmonary interactions, the patient’s underlying health status, elevated abdominal pressure, the use of cardioactive drugs, and strict drug control conditions [20]. Consequently, assessing the intravascular volume of obstetric patients with spontaneous respiration remains a challenging task [21].

As far as we know, evaluation of common carotid artery (CCA) blood flow can provide valuable information regarding the hemodynamic status of a patient. In the recent years, the carotid artery VTI as well as measures of their variation induced by the respiratory cycle, have demonstrated a direct correlation with aortic VTI and have been proposed as fast and easy to obtain ultrasound measures for assessing fluid responsiveness in intensive care unit patients [22]. Importantly, Sidor et al. confirmed that total carotid flow (TCF) calculated based on volume-time integral (VTI) in the carotid artery showed positive correlation to cardiac output and carotid systolic VTI was one of the most promising indicators to assess fluid responsiveness and help fluid management in hemodynamically stable participants [7]. Interestingly, during passive leg raising (PLR), an increase of the VTI of subaortic blood flow (ΔVTI) above 12% predicted the response with a sensitivity and specificity of 75 [95% confident interval (CI): 0.42–0.95] and 100% (95% CI: 0.72–1.00), respectively, ΔVTI combined with PLR could accurately predict fluid responsiveness in the specific setting of severe preeclampsia (SP) [23]. In addition, measurement of the subaortic variation in the velocity time integral (VTI) after passive leg raising allows prediction of fluid responsiveness [1]. A prospective observational study also confirmed the good agreement (Cohen’s kappa coefficient) between the carotid and aortic velocity-time integral (ΔVTI) of 0.84 (95% CI 0.68–0.99) and that between the carotid and aortic Doppler peak velocity (ΔDPV) of 0.76 (95% CI 0.88–0.94) [22]. Another recent prospective observational trial further demonstrated that the combined parameters of heart rate (HR) and left ventricular end-diastolic area (LVEDA)s with VTI% may predict spinal anaesthesia-induced hypotension more precisely than the single parameters in pregnant women undergoing combined spinal-epidural anaesthesia for elective caesarean Section [24].

In this context, our study showed that fluid challenge markedly increased VTI in both non-responders and responders groups and the area under the ROC curve to predict fluid responsiveness for VTI was 0.821, the sensitivity and specificity for carotid artery blood flow and VTI are 67%, 90%, with the cut-off values is 8.7 cm/s. Based on the above literatures and our findings, ultrasound measurements of VTI is displayed as an effective indices for predicting fluid responsiveness in pregnant women, suggesting that carotid artery VTI provides characterization of the risk of capacity overload or insufficient during elective caesarean section under spinal anaesthesia, and therefore may allow individualised strategies for prevention and management. Further work is needed to validate the correlations of ΔVTI and stroke volume (SV) and cardiac index (Ci) and utilize these acquired carotid parameters to guide fluid management and predict fluid responsiveness in pregnant women.

Up to now, emerging evidence showed that carotid blood flow measurements correlated moderately with cardiac output and may be a better marker of cardiac output and less subject to measurements issues than corrected carotid flow time [8]. Accordingly, carotid blood flow (CBF), which was calculated based on both systolic VTI and total VTI, correlated very strongly with SV, indicating that Doppler ultrasonography of the left common carotid artery (CCA) is able to estimate the SV and cardiac index (Ci) of critically ill children and therefore, the carotid Doppler ultrasonography may be considered as an alternative for estimating Ci when transthoracic echocardiography (TTE) is not feasible or available [25]. Of note, Gassner et al. found that intraclass correlation coefficient (ICC) analysis demonstrated almost perfect correlation (0.8152) between measurements of CO via ultrasound vs. invasive modalities, while the ICC between POCUS and the invasive measurement via arterial waveform pulse contour analysis was 0.84 and via the pulmonary artery catheter was 0.74, which showed a basic consistency between ultrasound and the two invasive devices and indicated that common carotid artery POCUS offers a non-invasive method of measuring the CO in the critically ill population [26].

As our results indicated, the predictability of carotid artery blood flow was comparable to that of carotid artery VTI with excellent interobserver agreement. Moreover, carotid artery blood flow yielded a cut-off value with the highest sensitivity and specificity. We also showed fluid challenge significantly increased carotid artery diameter and carotid artery blood flow in both two groups after the fluid challenge, suggesting their strong association with preload. Unfortunately, our findings were not consistent with previous study which stated that in patients with suspected sepsis, a fluid challenge did not result in a significant change in CBF, the reason may be due to the patient style and PLR [27]. In the present study, we comprehensively evaluated the ability of the carotid artery to predict volume responsiveness from both the carotid artery VTI and blood flow, which can provide more favourable evidence for the clinical use of the carotid artery in evaluating volume state. Thus, it remains to be verified through further studies and more clinical experience and identify the key limiting factors in using carotid ultrasound to determine fluid responsiveness.

Intravertebral anaesthesia is usually the first choice for caesarean section, and CSEA is characterized by rapid onset, low dosage of local anaesthesia, perfect analgesia and muscle relaxation effects, and no restriction of operation time, and has been widely used in clinic. Hypotension is a common complication of CSEA, and the incidence of hypotension after caesarean section CSEA is more than 80% without preventive management [28]. The main causes include extensive block of preganglionic fibers of sympathetic nerve, uterine compression of abdominal aorta and inferior vena cava, relaxation of abdominal muscles, principal ligaments of uterus and sacral ligaments after anaesthesia, fasting and drinking before anaesthesia, and changes of autonomic nerve balance during pregnancy [29]. Maternal blood pressure drop can cause different degrees of cerebral hypoperfusion, manifested as nausea, vomiting, chest tightness, dyspnoea, and even consciousness disturbance, circulatory arrest. Moreover, because the circulatory system of the uterus and placenta lacks its own regulatory function and the placental perfusion pressure depends on the maternal blood pressure, maternal hypotension can reduce the uterus and placenta perfusion, causing fatal distress-hypoxia, acidosis, hypercapnia, and even central nervous system damage [30]. At present, according to the mechanism of hypotension, a variety of prevention and treatment methods have been proposed in clinical practice, such as predilation before anaesthesia, body position changes, control of anaesthesia block plane, type of local anaesthesia drugs, use of 5-HT3 receptor antagonists, anticholinergics, and epidural anaesthesia, etc. [31, 32]. Interestingly, the usual prediction methods include the symptoms of supine position, the difference of blood pressure and heart rate between left and supine position, heart rate spectrum analysis, and pulse perfusion index, etc. [33, 34]. In our study, the total incidence of hypotension after CSEA of the two groups was decreased to 21.1%, suggested that real-time detecting and management of hypovolemia (fluid responsiveness) dramatically reduced the incidence of hypotension after anaesthesia, which were mainly due to the fluid challenge and fine fluid infusion. Currently, it is believed that the effect of using one method alone during caesarean section to avoid hypotension is not enough, and thus it is necessary to reasonably evaluate and predict the mother’s own situation before surgery, and select a variety of prevention and treatment methods in combination, among which the use of small doses of local anaesthesia to perform CSEA, and the maintenance of circulation stability while ensuring the aesthetic effect are fundamental. Our finding is helpful for clinical practice, and further research is needed to assess the techniques used to predict hypotension after CSEA and is necessary to determine whether this knowledge improves maternal and neonatal outcomes.

This study has some limitations. First of all, in our study, only 72 women who chose elective caesarean section were enrolled. We will recruit more obstetric patients to explore the best ultrasound technique and the cut-off points for predicting fluid responsiveness and avoid overestimating the predictive power of these indices in our future studies. Second, this study was not conducted in women with gestational hypertension, preeclampsia, or emergency caesarean section, who are currently considered to be at high risk for hemodynamic instability. Future studies will determine the reliability and feasibility of ultrasonic techniques in predicting fluid responsiveness in pregnant women. Third, carotid artery blood flow and VTI are negatively correlated with systemic vascular resistance and are affected by left ventricular preload and myocardial strength. Therefore, many other factors that alter afterload also affect VTI and carotid artery blood flow^7^. In future studies, the predictive power of VTI and carotid blood flow in different populations and clinical settings should be evaluated. Fourth, in this study, ultrasound technology was only applied to perioperative parturients to accurately measure carotid artery blood flow and VTI to predict fluid responsiveness. Future studies will use ultrasound measurements of other peripheral arteries, such as the radial or brachial arteries, to predict fluid reactivity in pregnant women. Our study demonstrated that ultrasound evaluation of VTI and carotid artery blood flow appeared to be the accurate indicators of fluid responsiveness in pregnant women. Future research should focus further on the accuracy and reliability of carotid artery blood flow and VTI, as well as their relations to other sonographic predictive measurements.

## Conclusions

Our study addressed that ultrasound measurement of CABF and VTI appeared to be the valuable predictors of fluid responsiveness in spontaneously breathing parturients undergoing elective caesarean section and detecting and management of hypovolemia (fluid responsiveness) could significantly decrease the incidence of hypotension after anaesthesia. Our findings contribute to the understanding of maternal volume status and fluid management in obstetric anaesthesia and are expected to provide the new ultrasound evaluation methods for predicting maternal fluid reactivity. Further work is needed to validate these correlations between maternal volume status and different hemodynamic parameters in the CCA, namely CABF and VTI and utilize these acquired carotid parameters to guide fluid management and predict fluid responsiveness.

## Data Availability

The datasets used and/or analysed during the current study available from the corresponding author on reasonable request.

## Abbreviations

CABF: Carotid artery blood flow
VTI: Velocity time integral
SVI: Stroke volume index
SV: Stroke volume
CO: Cardiac output
ROC: The receiver operating characteristic
CI: Confidence interval
IVCCI: Inferior vena cava collapsibility index
ASA: American Society of Anaesthesiologists
BSA: Body surface area
AUC: Area under the curve
CV: Coefficient of variation
ICC: Intraclass correlation coefficient
LOA: Limits of agreement
CCA: Common carotid artery
TCF: Total carotid flow
PLR: Passive leg raising
Ci: Cardiac index
CBF: Carotid blood flow
TTE: Transthoracic echocardiography
ICC: Intraclass correlation coefficient
CSEA: Combined spinal-epidural anaesthesia
